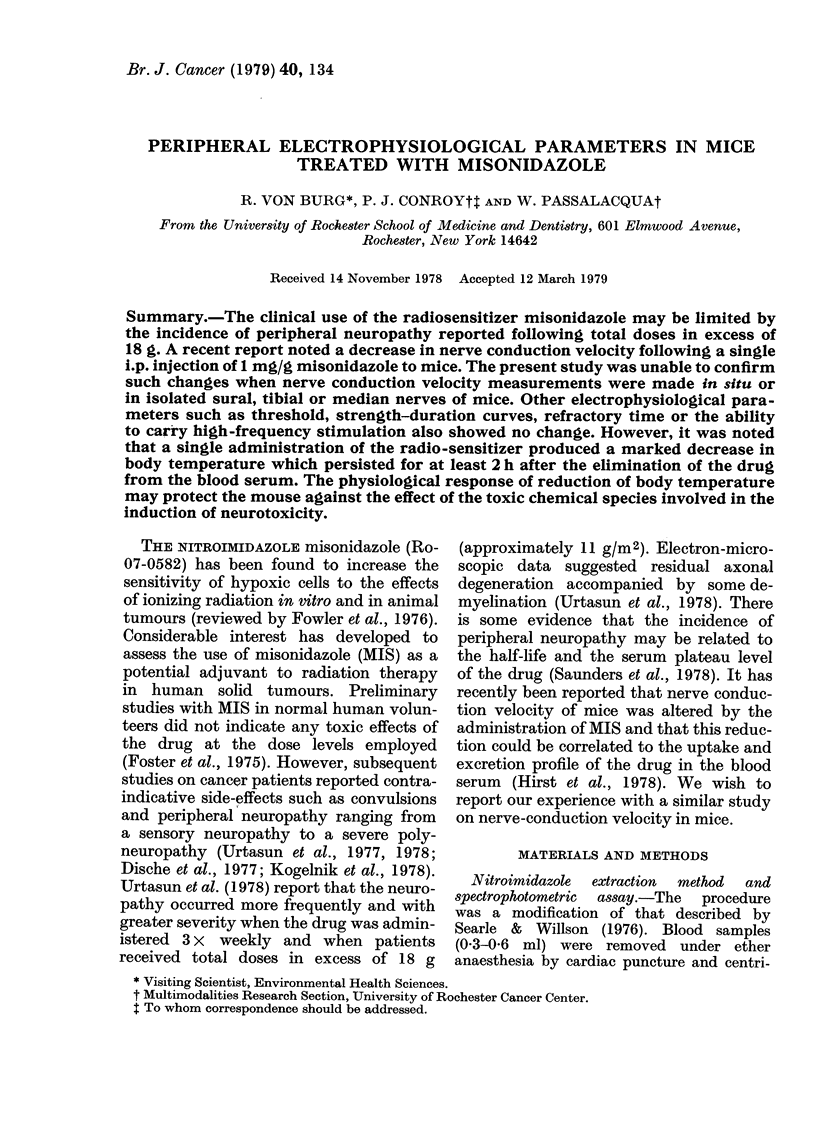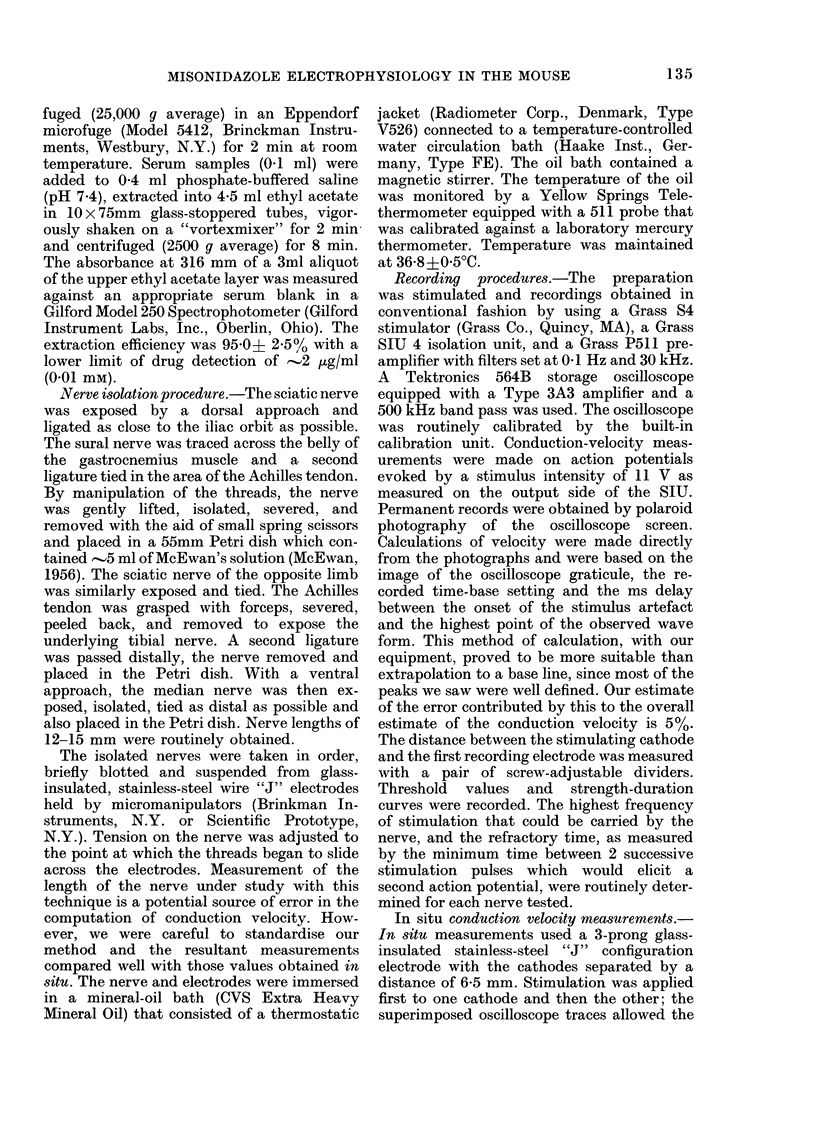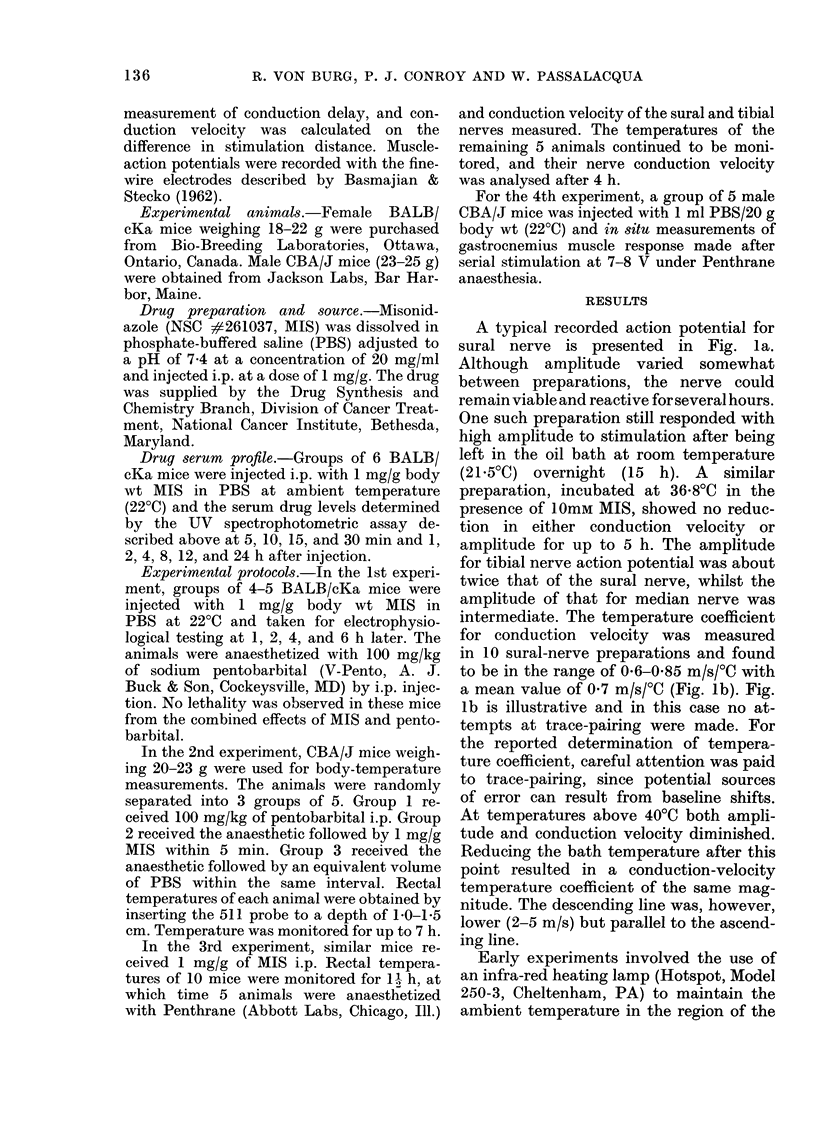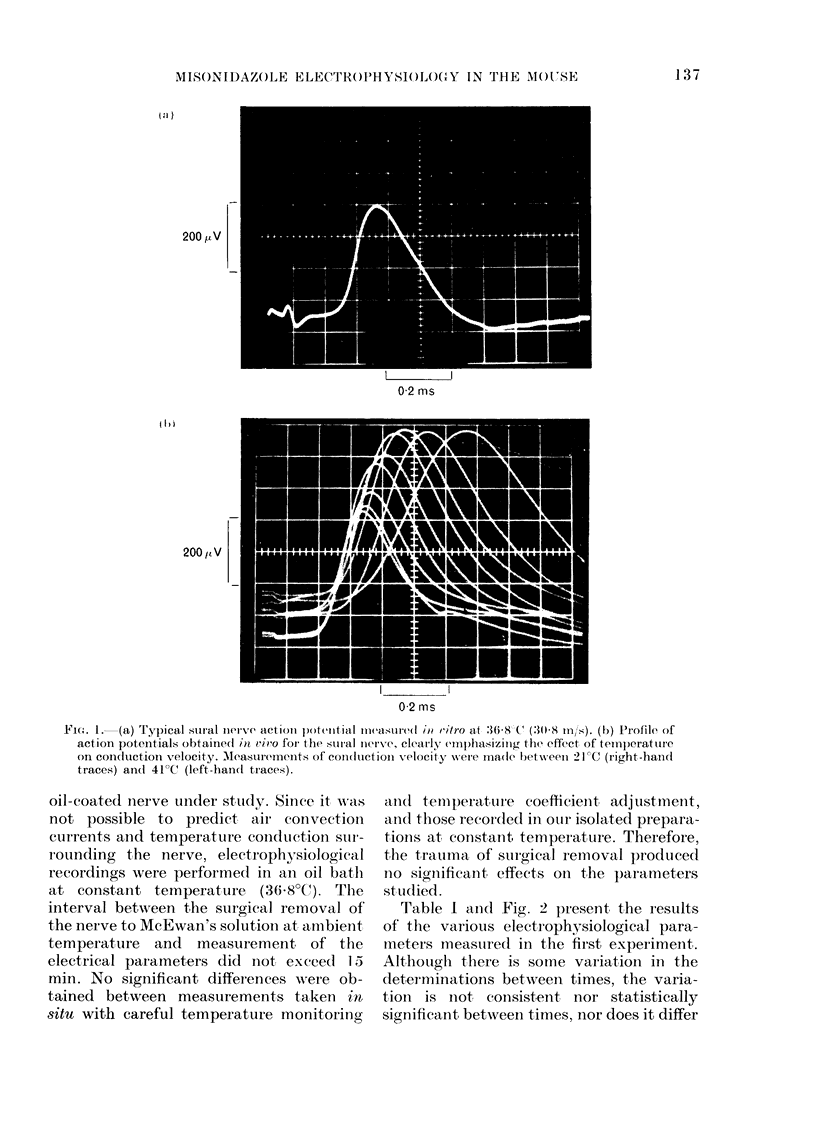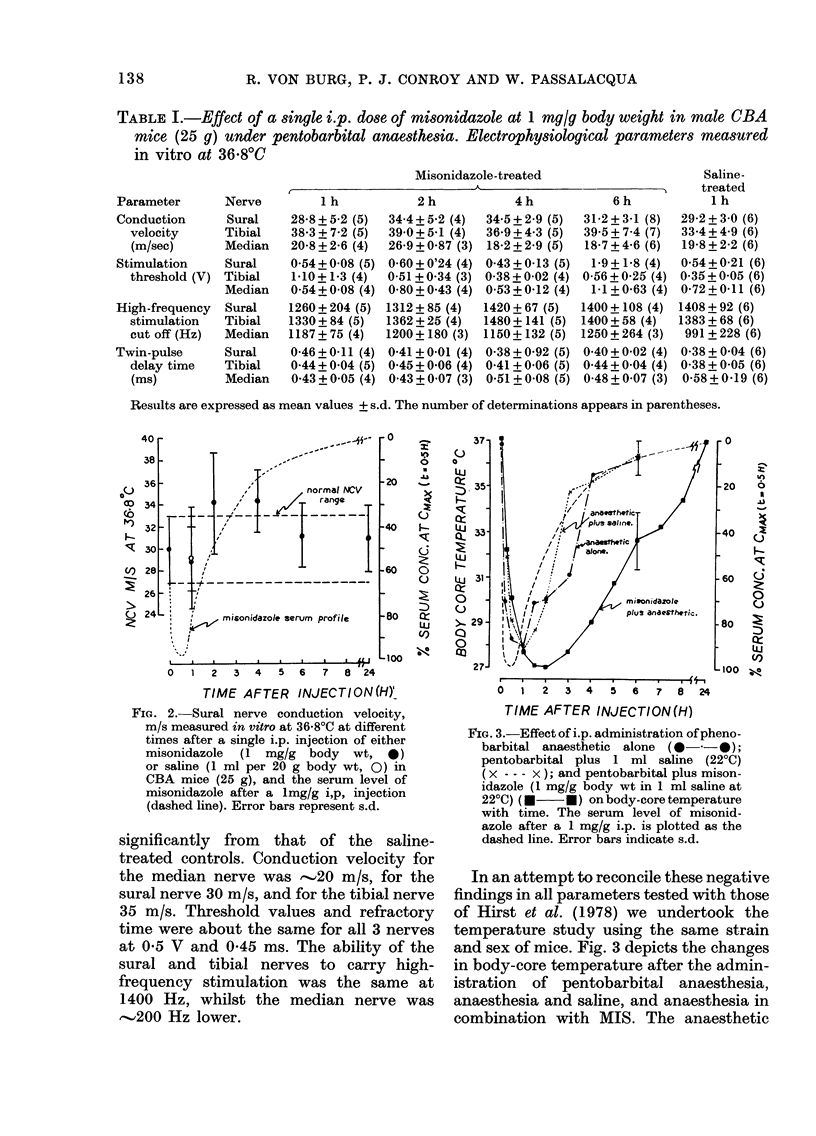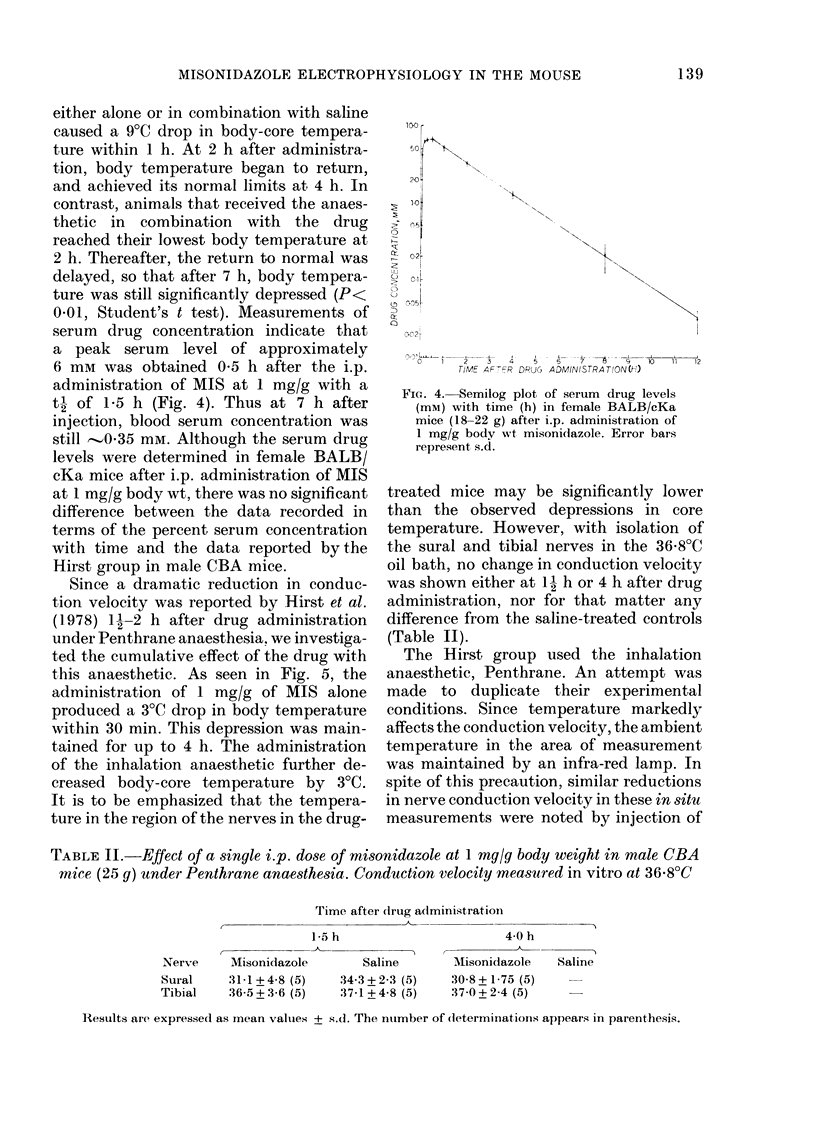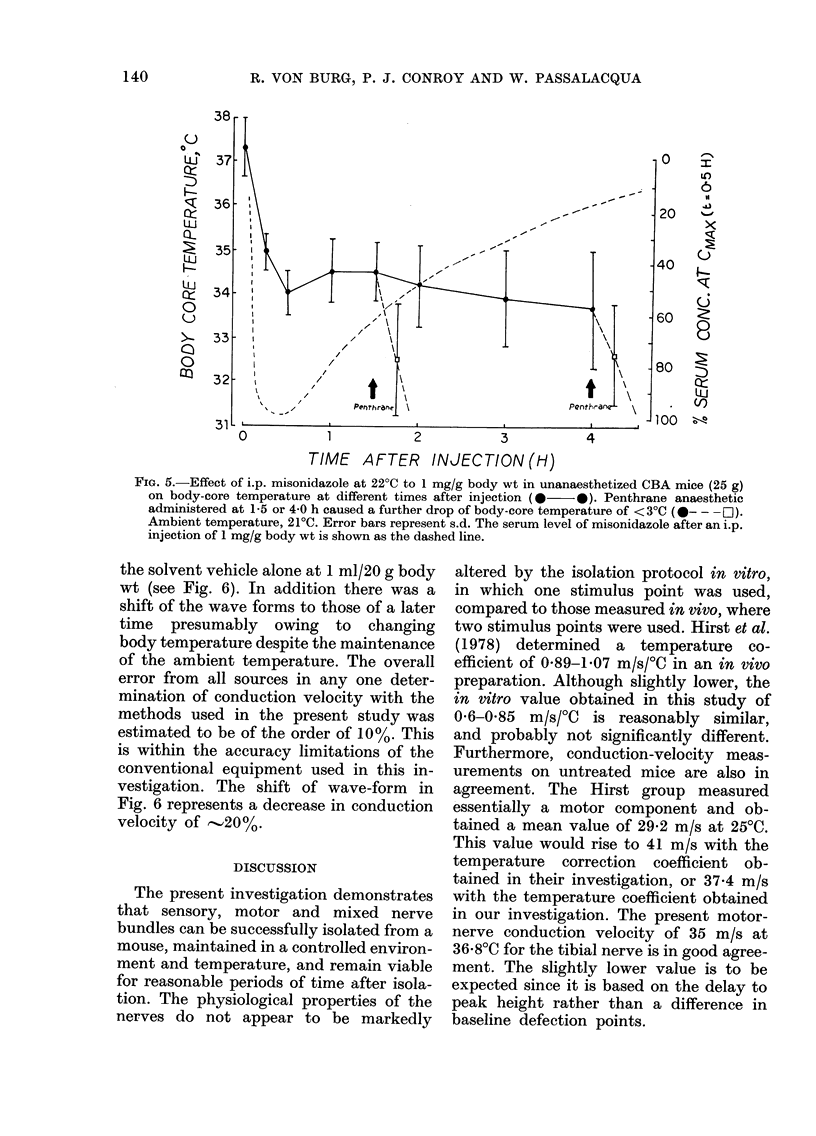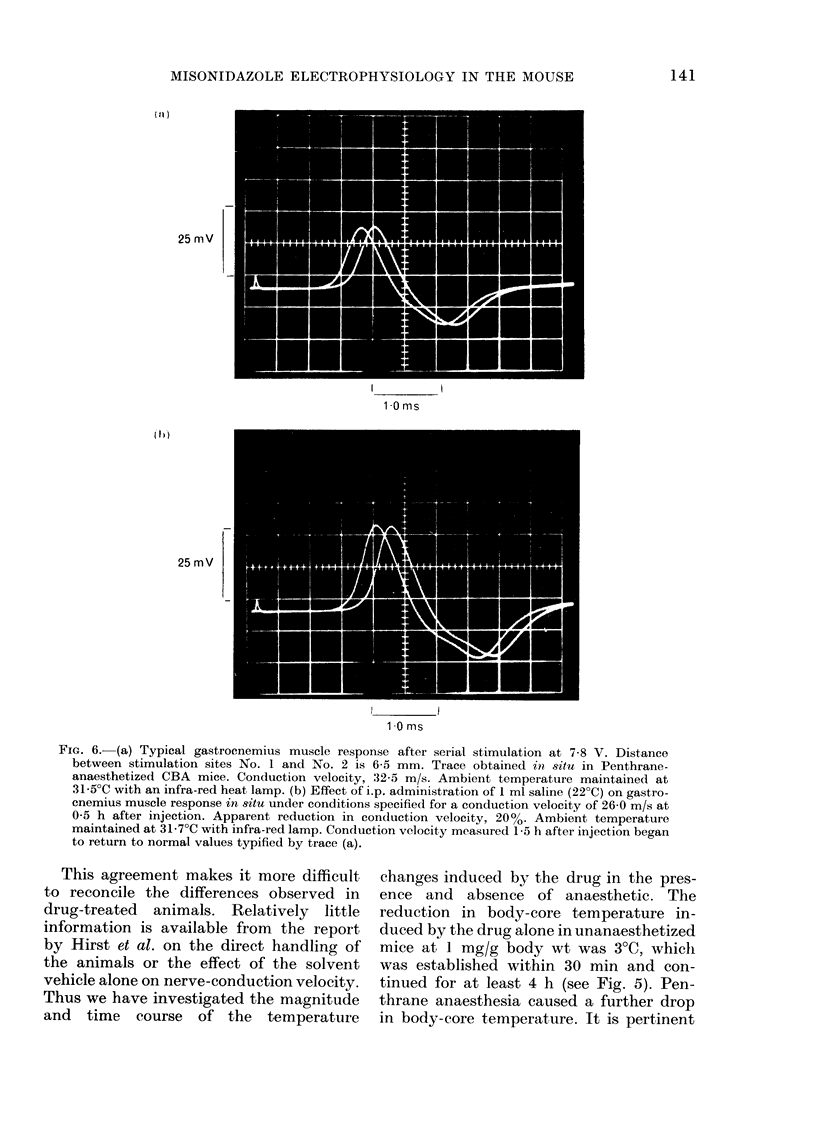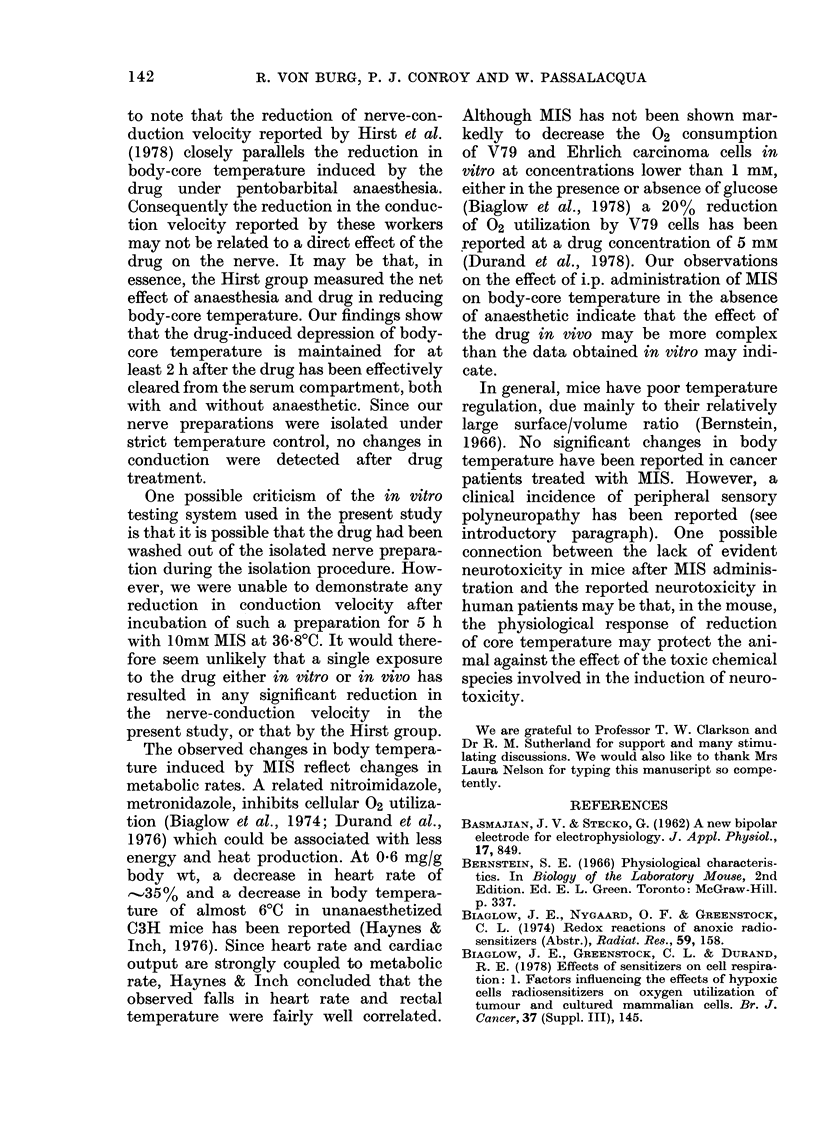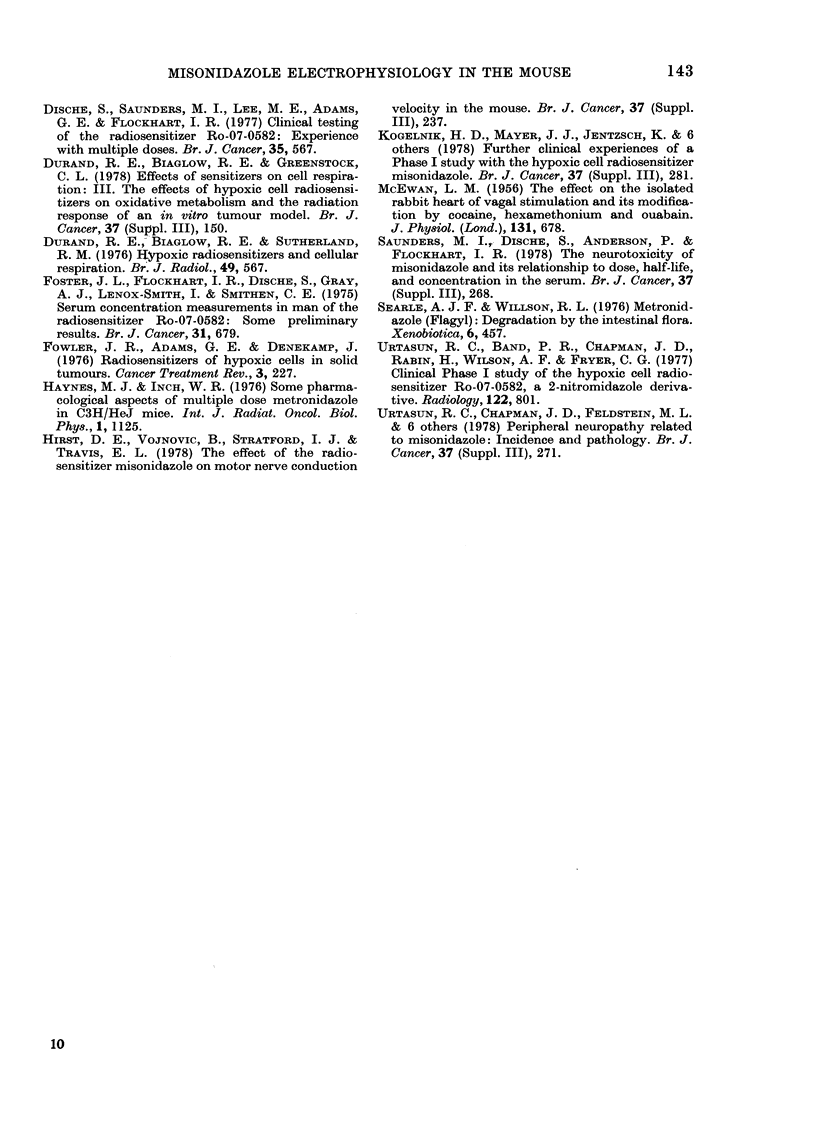# Peripheral electrophysiological parameters in mice treated with misonidazole.

**DOI:** 10.1038/bjc.1979.149

**Published:** 1979-07

**Authors:** R. Von Burg, P. J. Conroy, W. Passalacqua

## Abstract

**Images:**


*Br. J. Cancer* (1979) **40**, 134

### PERIPHERAL ELECTROPHYSIOLOGICAL PARAMETERS IN MICE TREATED WITH MISONIDAZOLE

R. VON BURG*, P. J. CONROY†‡ AND W. PASSALACQUA†

*From the University of Rochester School of Medicine and Dentistry*, 601 *Elmwood Avenue, Rochester, New York* 14642

Received 14 November 1978 Accepted 12 March 1979

**Summary.—The clinical use of the radiosensitizer misonidazole may be limited by the incidence of peripheral neuropathy reported following total doses in excess of 18 g. A recent report noted a decrease in nerve conduction velocity following a single i.p. injection of 1 mg/g misonidazole to mice. The present study was unable to confirm such changes when nerve conduction velocity measurements were made *in situ* or in isolated sural, tibial or median nerves of mice. Other electrophysiological parameters such as threshold, strength–duration curves, refractory time or the ability to carry high-frequency stimulation also showed no change. However, it was noted that a single administration of the radio-sensitizer produced a marked decrease in body temperature which persisted for at least 2 h after the elimination of the drug from the blood serum. The physiological response of reduction of body temperature may protect the mouse against the effect of the toxic chemical species involved in the induction of neurotoxicity.**

THE NITROIMIDAZOLE misonidazole (Ro-07-0582) has been found to increase the sensitivity of hypoxic cells to the effects of ionizing radiation *in vitro* and in animal tumours (reviewed by Fowler *et al.*, 1976). Considerable interest has developed to assess the use of misonidazole (MIS) as a potential adjuvant to radiation therapy in human solid tumours. Preliminary studies with MIS in normal human volunteers did not indicate any toxic effects of the drug at the dose levels employed (Foster *et al.*, 1975). However, subsequent studies on cancer patients reported contra-indicative side-effects such as convulsions and peripheral neuropathy ranging from a sensory neuropathy to a severe poly-neuropathy (Urtasun *et al.*, 1977, 1978; Dische *et al.*, 1977; Kogelnik *et al.*, 1978). Urtasun *et al.* (1978) report that the neuropathy occurred more frequently and with greater severity when the drug was administered 3× weekly and when patients received total doses in excess of 18 g (approximately 11 g/m$^2$). Electron-microscopic data suggested residual axonal degeneration accompanied by some de-myelination (Urtasun *et al.*, 1978). There is some evidence that the incidence of peripheral neuropathy may be related to the half-life and the serum plateau level of the drug (Saunders *et al.*, 1978). It has recently been reported that nerve conduction velocity of mice was altered by the administration of MIS and that this reduction could be correlated to the uptake and excretion profile of the drug in the blood serum (Hirst *et al.*, 1978). We wish to report our experience with a similar study on nerve-conduction velocity in mice.

### MATERIALS AND METHODS

*Nitroimidazole extraction method and spectrophotometric assay.*—The procedure was a modification of that described by Searle & Willson (1976). Blood samples (0·3–0·6 ml) were removed under ether anaesthesia by cardiac puncture and centri-

* Visiting Scientist, Environmental Health Sciences.
† Multimodalities Research Section, University of Rochester Cancer Center.
‡ To whom correspondence should be addressed.

fuged (25,000 *g* average) in an Eppendorf microfuge (Model 5412, Brinckman Instruments, Westbury, N.Y.) for 2 min at room temperature. Serum samples (0·1 ml) were added to 0·4 ml phosphate-buffered saline (pH 7·4), extracted into 4·5 ml ethyl acetate in 10×75mm glass-stoppered tubes, vigorously shaken on a "vortexmixer" for 2 min and centrifuged (2500 *g* average) for 8 min. The absorbance at 316 mm of a 3ml aliquot of the upper ethyl acetate layer was measured against an appropriate serum blank in a Gilford Model 250 Spectrophotometer (Gilford Instrument Labs, Inc., Oberlin, Ohio). The extraction efficiency was 95·0± 2·5% with a lower limit of drug detection of ~2 μg/ml (0·01 mM).

*Nerve isolation procedure.*—The sciatic nerve was exposed by a dorsal approach and ligated as close to the iliac orbit as possible. The sural nerve was traced across the belly of the gastrocnemius muscle and a second ligature tied in the area of the Achilles tendon. By manipulation of the threads, the nerve was gently lifted, isolated, severed, and removed with the aid of small spring scissors and placed in a 55mm Petri dish which contained ~5 ml of McEwan's solution (McEwan, 1956). The sciatic nerve of the opposite limb was similarly exposed and tied. The Achilles tendon was grasped with forceps, severed, peeled back, and removed to expose the underlying tibial nerve. A second ligature was passed distally, the nerve removed and placed in the Petri dish. With a ventral approach, the median nerve was then exposed, isolated, tied as distal as possible and also placed in the Petri dish. Nerve lengths of 12–15 mm were routinely obtained.

The isolated nerves were taken in order, briefly blotted and suspended from glass-insulated, stainless-steel wire "J" electrodes held by micromanipulators (Brinkman Instruments, N.Y. or Scientific Prototype, N.Y.). Tension on the nerve was adjusted to the point at which the threads began to slide across the electrodes. Measurement of the length of the nerve under study with this technique is a potential source of error in the computation of conduction velocity. However, we were careful to standardise our method and the resultant measurements compared well with those values obtained *in situ*. The nerve and electrodes were immersed in a mineral-oil bath (CVS Extra Heavy Mineral Oil) that consisted of a thermostatic jacket (Radiometer Corp., Denmark, Type V526) connected to a temperature-controlled water circulation bath (Haake Inst., Germany, Type FE). The oil bath contained a magnetic stirrer. The temperature of the oil was monitored by a Yellow Springs Telethermometer equipped with a 511 probe that was calibrated against a laboratory mercury thermometer. Temperature was maintained at 36·8±0·5°C.

*Recording procedures.*—The preparation was stimulated and recordings obtained in conventional fashion by using a Grass S4 stimulator (Grass Co., Quincy, MA), a Grass SIU 4 isolation unit, and a Grass P511 preamplifier with filters set at 0·1 Hz and 30 kHz. A Tektronics 564B storage oscilloscope equipped with a Type 3A3 amplifier and a 500 kHz band pass was used. The oscilloscope was routinely calibrated by the built-in calibration unit. Conduction-velocity measurements were made on action potentials evoked by a stimulus intensity of 11 V as measured on the output side of the SIU. Permanent records were obtained by polaroid photography of the oscilloscope screen. Calculations of velocity were made directly from the photographs and were based on the image of the oscilloscope graticule, the recorded time-base setting and the ms delay between the onset of the stimulus artefact and the highest point of the observed wave form. This method of calculation, with our equipment, proved to be more suitable than extrapolation to a base line, since most of the peaks we saw were well defined. Our estimate of the error contributed by this to the overall estimate of the conduction velocity is 5%. The distance between the stimulating cathode and the first recording electrode was measured with a pair of screw-adjustable dividers. Threshold values and strength-duration curves were recorded. The highest frequency of stimulation that could be carried by the nerve, and the refractory time, as measured by the minimum time between 2 successive stimulation pulses which would elicit a second action potential, were routinely determined for each nerve tested.

In situ *conduction velocity measurements.*—*In situ* measurements used a 3-prong glass-insulated stainless-steel "J" configuration electrode with the cathodes separated by a distance of 6·5 mm. Stimulation was applied first to one cathode and then the other; the superimposed oscilloscope traces allowed the measurement of conduction delay, and conduction velocity was calculated on the difference in stimulation distance. Muscle-action potentials were recorded with the fine-wire electrodes described by Basmajian & Stecko (1962).

*Experimental animals.*—Female BALB/cKa mice weighing 18–22 g were purchased from Bio-Breeding Laboratories, Ottawa, Ontario, Canada. Male CBA/J mice (23–25 g) were obtained from Jackson Labs, Bar Harbor, Maine.

*Drug preparation and source.*—Misonidazole (NSC #261037, MIS) was dissolved in phosphate-buffered saline (PBS) adjusted to a pH of 7·4 at a concentration of 20 mg/ml and injected i.p. at a dose of 1 mg/g. The drug was supplied by the Drug Synthesis and Chemistry Branch, Division of Cancer Treatment, National Cancer Institute, Bethesda, Maryland.

*Drug serum profile.*—Groups of 6 BALB/cKa mice were injected i.p. with 1 mg/g body wt MIS in PBS at ambient temperature (22°C) and the serum drug levels determined by the UV spectrophotometric assay described above at 5, 10, 15, and 30 min and 1, 2, 4, 8, 12, and 24 h after injection.

*Experimental protocols.*—In the 1st experiment, groups of 4–5 BALB/cKa mice were injected with 1 mg/g body wt MIS in PBS at 22°C and taken for electrophysiological testing at 1, 2, 4, and 6 h later. The animals were anaesthetized with 100 mg/kg of sodium pentobarbital (V-Pento, A. J. Buck & Son, Cockeysville, MD) by i.p. injection. No lethality was observed in these mice from the combined effects of MIS and pentobarbital.

In the 2nd experiment, CBA/J mice weighing 20–23 g were used for body-temperature measurements. The animals were randomly separated into 3 groups of 5. Group 1 received 100 mg/kg of pentobarbital i.p. Group 2 received the anaesthetic followed by 1 mg/g MIS within 5 min. Group 3 received the anaesthetic followed by an equivalent volume of PBS within the same interval. Rectal temperatures of each animal were obtained by inserting the 511 probe to a depth of 1·0–1·5 cm. Temperature was monitored for up to 7 h.

In the 3rd experiment, similar mice received 1 mg/g of MIS i.p. Rectal temperatures of 10 mice were monitored for $1\frac{1}{2}$ h, at which time 5 animals were anaesthetized with Penthrane (Abbott Labs, Chicago, Ill.) and conduction velocity of the sural and tibial nerves measured. The temperatures of the remaining 5 animals continued to be monitored, and their nerve conduction velocity was analysed after 4 h.

For the 4th experiment, a group of 5 male CBA/J mice was injected with 1 ml PBS/20 g body wt (22°C) and *in situ* measurements of gastrocnemius muscle response made after serial stimulation at 7–8 V under Penthrane anaesthesia.

### RESULTS

A typical recorded action potential for sural nerve is presented in Fig. 1a. Although amplitude varied somewhat between preparations, the nerve could remain viable and reactive for several hours. One such preparation still responded with high amplitude to stimulation after being left in the oil bath at room temperature (21·5°C) overnight (15 h). A similar preparation, incubated at 36·8°C in the presence of 10mM MIS, showed no reduction in either conduction velocity or amplitude for up to 5 h. The amplitude for tibial nerve action potential was about twice that of the sural nerve, whilst the amplitude of that for median nerve was intermediate. The temperature coefficient for conduction velocity was measured in 10 sural-nerve preparations and found to be in the range of 0·6–0·85 m/s/°C with a mean value of 0·7 m/s/°C (Fig. 1b). Fig. 1b is illustrative and in this case no attempts at trace-pairing were made. For the reported determination of temperature coefficient, careful attention was paid to trace-pairing, since potential sources of error can result from baseline shifts. At temperatures above 40°C both amplitude and conduction velocity diminished. Reducing the bath temperature after this point resulted in a conduction-velocity temperature coefficient of the same magnitude. The descending line was, however, lower (2–5 m/s) but parallel to the ascending line.

Early experiments involved the use of an infra-red heating lamp (Hotspot, Model 250-3, Cheltenham, PA) to maintain the ambient temperature in the region of the

FIG. 1.—(a) Typical sural nerve action potential measured *in vitro* at 36·8°C (30·8 m/s). (b) Profile of action potentials obtained *in vivo* for the sural nerve, clearly emphasizing the effect of temperature on conduction velocity. Measurements of conduction velocity were made between 21°C (right-hand traces) and 41°C (left-hand traces).

oil-coated nerve under study. Since it was not possible to predict air convection currents and temperature conduction surrounding the nerve, electrophysiological recordings were performed in an oil bath at constant temperature (36·8°C). The interval between the surgical removal of the nerve to McEwan's solution at ambient temperature and measurement of the electrical parameters did not exceed 15 min. No significant differences were obtained between measurements taken *in situ* with careful temperature monitoring and temperature coefficient adjustment, and those recorded in our isolated preparations at constant temperature. Therefore, the trauma of surgical removal produced no significant effects on the parameters studied.

Table I and Fig. 2 present the results of the various electrophysiological parameters measured in the first experiment. Although there is some variation in the determinations between times, the variation is not consistent nor statistically significant between times, nor does it differ

TABLE I.—*Effect of a single i.p. dose of misonidazole at 1 mg/g body weight in male CBA mice (25 g) under pentobarbital anaesthesia. Electrophysiological parameters measured* in vitro *at 36·8°C*

| | | Misonidazole-treated | | | | Saline-treated |
|---|---|---|---|---|---|---|
| Parameter | Nerve | 1 h | 2 h | 4 h | 6 h | 1 h |
| Conduction velocity (m/sec) | Sural | 28·8 ± 5·2 (5) | 34·4 ± 5·2 (4) | 34·5 ± 2·9 (5) | 31·2 ± 3·1 (8) | 29·2 ± 3·0 (6) |
| | Tibial | 38·3 ± 7·2 (5) | 39·0 ± 5·1 (4) | 36·9 ± 4·3 (5) | 39·5 ± 7·4 (7) | 33·4 ± 4·9 (6) |
| | Median | 20·8 ± 2·6 (4) | 26·9 ± 0·87 (3) | 18·2 ± 2·9 (5) | 18·7 ± 4·6 (6) | 19·8 ± 2·2 (6) |
| Stimulation threshold (V) | Sural | 0·54 ± 0·08 (5) | 0·60 ± 0’24 (4) | 0·43 ± 0·13 (5) | 1·9 ± 1·8 (4) | 0·54 ± 0·21 (6) |
| | Tibial | 1·10 ± 1·3 (4) | 0·51 ± 0·34 (3) | 0·38 ± 0·02 (4) | 0·56 ± 0·25 (4) | 0·35 ± 0·05 (6) |
| | Median | 0·54 ± 0·08 (4) | 0·80 ± 0·43 (4) | 0·53 ± 0·12 (4) | 1·1 ± 0·63 (4) | 0·72 ± 0·11 (6) |
| High-frequency stimulation cut off (Hz) | Sural | 1260 ± 204 (5) | 1312 ± 85 (4) | 1420 ± 67 (5) | 1400 ± 108 (4) | 1408 ± 92 (6) |
| | Tibial | 1330 ± 84 (5) | 1362 ± 25 (4) | 1480 ± 141 (5) | 1400 ± 58 (4) | 1383 ± 68 (6) |
| | Median | 1187 ± 75 (4) | 1200 ± 180 (3) | 1150 ± 132 (5) | 1250 ± 264 (3) | 991 ± 228 (6) |
| Twin-pulse delay time (ms) | Sural | 0·46 ± 0·11 (4) | 0·41 ± 0·01 (4) | 0·38 ± 0·92 (5) | 0·40 ± 0·02 (4) | 0·38 ± 0·04 (6) |
| | Tibial | 0·44 ± 0·04 (5) | 0·45 ± 0·06 (4) | 0·41 ± 0·06 (5) | 0·44 ± 0·04 (4) | 0·38 ± 0·05 (6) |
| | Median | 0·43 ± 0·05 (4) | 0·43 ± 0·07 (3) | 0·51 ± 0·08 (5) | 0·48 ± 0·07 (3) | 0·58 ± 0·19 (6) |

Results are expressed as mean values ± s.d. The number of determinations appears in parentheses.

FIG. 2.—Sural nerve conduction velocity, m/s measured *in vitro* at 36·8°C at different times after a single i.p. injection of either misonidazole (1 mg/g body wt, ●) or saline (1 ml per 20 g body wt, ○) in CBA mice (25 g), and the serum level of misonidazole after a 1mg/g i,p, injection (dashed line). Error bars represent s.d.

FIG. 3.—Effect of i.p. administration of phenobarbital anaesthetic alone (●—·—●); pentobarbital plus 1 ml saline (22°C) (× - - - ×); and pentobarbital plus misonidazole (1 mg/g body wt in 1 ml saline at 22°C) (■——■) on body-core temperature with time. The serum level of misonidazole after a 1 mg/g i.p. is plotted as the dashed line. Error bars indicate s.d.

significantly from that of the saline-treated controls. Conduction velocity for the median nerve was ~20 m/s, for the sural nerve 30 m/s, and for the tibial nerve 35 m/s. Threshold values and refractory time were about the same for all 3 nerves at 0·5 V and 0·45 ms. The ability of the sural and tibial nerves to carry high-frequency stimulation was the same at 1400 Hz, whilst the median nerve was ~200 Hz lower.

In an attempt to reconcile these negative findings in all parameters tested with those of Hirst *et al.* (1978) we undertook the temperature study using the same strain and sex of mice. Fig. 3 depicts the changes in body-core temperature after the administration of pentobarbital anaesthesia, anaesthesia and saline, and anaesthesia in combination with MIS. The anaesthetic either alone or in combination with saline caused a 9°C drop in body-core temperature within 1 h. At 2 h after administration, body temperature began to return, and achieved its normal limits at 4 h. In contrast, animals that received the anaesthetic in combination with the drug reached their lowest body temperature at 2 h. Thereafter, the return to normal was delayed, so that after 7 h, body temperature was still significantly depressed ($P<0{\cdot}01$, Student's $t$ test). Measurements of serum drug concentration indicate that a peak serum level of approximately 6 mM was obtained 0·5 h after the i.p. administration of MIS at 1 mg/g with a $t_{\frac{1}{2}}$ of 1·5 h (Fig. 4). Thus at 7 h after injection, blood serum concentration was still ~0·35 mM. Although the serum drug levels were determined in female BALB/cKa mice after i.p. administration of MIS at 1 mg/g body wt, there was no significant difference between the data recorded in terms of the percent serum concentration with time and the data reported by the Hirst group in male CBA mice.

Fig. 4.—Semilog plot of serum drug levels (mM) with time (h) in female BALB/cKa mice (18–22 g) after i.p. administration of 1 mg/g body wt misonidazole. Error bars represent s.d.

Since a dramatic reduction in conduction velocity was reported by Hirst *et al.* (1978) $1\frac{1}{2}$–2 h after drug administration under Penthrane anaesthesia, we investigated the cumulative effect of the drug with this anaesthetic. As seen in Fig. 5, the administration of 1 mg/g of MIS alone produced a 3°C drop in body temperature within 30 min. This depression was maintained for up to 4 h. The administration of the inhalation anaesthetic further decreased body-core temperature by 3°C. It is to be emphasized that the temperature in the region of the nerves in the drug-treated mice may be significantly lower than the observed depressions in core temperature. However, with isolation of the sural and tibial nerves in the 36·8°C oil bath, no change in conduction velocity was shown either at $1\frac{1}{2}$ h or 4 h after drug administration, nor for that matter any difference from the saline-treated controls (Table II).

The Hirst group used the inhalation anaesthetic, Penthrane. An attempt was made to duplicate their experimental conditions. Since temperature markedly affects the conduction velocity, the ambient temperature in the area of measurement was maintained by an infra-red lamp. In spite of this precaution, similar reductions in nerve conduction velocity in these *in situ* measurements were noted by injection of

Table II.—*Effect of a single i.p. dose of misonidazole at* 1 *mg/g body weight in male CBA mice* (25 *g*) *under Penthrane anaesthesia. Conduction velocity measured* in vitro *at* 36·8°C

| | Time after drug administration | | | |
|---|---|---|---|---|
| | 1·5 h | | 4·0 h | |
| Nerve | Misonidazole | Saline | Misonidazole | Saline |
| Sural | 31·1 ± 4·8 (5) | 34·3 ± 2·3 (5) | 30·8 ± 1·75 (5) | — |
| Tibial | 36·5 ± 3·6 (5) | 37·1 ± 4·8 (5) | 37·0 ± 2·4 (5) | — |

Results are expressed as mean values ± s.d. The number of determinations appears in parenthesis.

FIG. 5.—Effect of i.p. misonidazole at 22°C to 1 mg/g body wt in unanaesthetized CBA mice (25 g) on body-core temperature at different times after injection (●——●). Penthrane anaesthetic administered at 1·5 or 4·0 h caused a further drop of body-core temperature of $<3$°C (●- - -□). Ambient temperature, 21°C. Error bars represent s.d. The serum level of misonidazole after an i.p. injection of 1 mg/g body wt is shown as the dashed line.

the solvent vehicle alone at 1 ml/20 g body wt (see Fig. 6). In addition there was a shift of the wave forms to those of a later time presumably owing to changing body temperature despite the maintenance of the ambient temperature. The overall error from all sources in any one determination of conduction velocity with the methods used in the present study was estimated to be of the order of 10%. This is within the accuracy limitations of the conventional equipment used in this investigation. The shift of wave-form in Fig. 6 represents a decrease in conduction velocity of ~20%.

### DISCUSSION

The present investigation demonstrates that sensory, motor and mixed nerve bundles can be successfully isolated from a mouse, maintained in a controlled environment and temperature, and remain viable for reasonable periods of time after isolation. The physiological properties of the nerves do not appear to be markedly altered by the isolation protocol *in vitro*, in which one stimulus point was used, compared to those measured *in vivo*, where two stimulus points were used. Hirst *et al.* (1978) determined a temperature coefficient of 0·89–1·07 m/s/°C in an *in vivo* preparation. Although slightly lower, the *in vitro* value obtained in this study of 0·6–0·85 m/s/°C is reasonably similar, and probably not significantly different. Furthermore, conduction-velocity measurements on untreated mice are also in agreement. The Hirst group measured essentially a motor component and obtained a mean value of 29·2 m/s at 25°C. This value would rise to 41 m/s with the temperature correction coefficient obtained in their investigation, or 37·4 m/s with the temperature coefficient obtained in our investigation. The present motor-nerve conduction velocity of 35 m/s at 36·8°C for the tibial nerve is in good agreement. The slightly lower value is to be expected since it is based on the delay to peak height rather than a difference in baseline defection points.

FIG. 6.—(a) Typical gastrocnemius muscle response after serial stimulation at 7·8 V. Distance between stimulation sites No. 1 and No. 2 is 6·5 mm. Trace obtained *in situ* in Penthrane-anaesthetized CBA mice. Conduction velocity, 32·5 m/s. Ambient temperature maintained at 31·5°C with an infra-red heat lamp. (b) Effect of i.p. administration of 1 ml saline (22°C) on gastrocnemius muscle response *in situ* under conditions specified for a conduction velocity of 26·0 m/s at 0·5 h after injection. Apparent reduction in conduction velocity, 20%. Ambient temperature maintained at 31·7°C with infra-red lamp. Conduction velocity measured 1·5 h after injection began to return to normal values typified by trace (a).

This agreement makes it more difficult to reconcile the differences observed in drug-treated animals. Relatively little information is available from the report by Hirst *et al.* on the direct handling of the animals or the effect of the solvent vehicle alone on nerve-conduction velocity. Thus we have investigated the magnitude and time course of the temperature changes induced by the drug in the presence and absence of anaesthetic. The reduction in body-core temperature induced by the drug alone in unanaesthetized mice at 1 mg/g body wt was 3°C, which was established within 30 min and continued for at least 4 h (see Fig. 5). Penthrane anaesthesia caused a further drop in body-core temperature. It is pertinent to note that the reduction of nerve-conduction velocity reported by Hirst *et al.* (1978) closely parallels the reduction in body-core temperature induced by the drug under pentobarbital anaesthesia. Consequently the reduction in the conduction velocity reported by these workers may not be related to a direct effect of the drug on the nerve. It may be that, in essence, the Hirst group measured the net effect of anaesthesia and drug in reducing body-core temperature. Our findings show that the drug-induced depression of body-core temperature is maintained for at least 2 h after the drug has been effectively cleared from the serum compartment, both with and without anaesthetic. Since our nerve preparations were isolated under strict temperature control, no changes in conduction were detected after drug treatment.

One possible criticism of the *in vitro* testing system used in the present study is that it is possible that the drug had been washed out of the isolated nerve preparation during the isolation procedure. However, we were unable to demonstrate any reduction in conduction velocity after incubation of such a preparation for 5 h with 10mM MIS at 36·8°C. It would therefore seem unlikely that a single exposure to the drug either *in vitro* or *in vivo* has resulted in any significant reduction in the nerve-conduction velocity in the present study, or that by the Hirst group.

The observed changes in body temperature induced by MIS reflect changes in metabolic rates. A related nitroimidazole, metronidazole, inhibits cellular $O_2$ utilization (Biaglow *et al.*, 1974; Durand *et al.*, 1976) which could be associated with less energy and heat production. At 0·6 mg/g body wt, a decrease in heart rate of ~35% and a decrease in body temperature of almost 6°C in unanaesthetized C3H mice has been reported (Haynes & Inch, 1976). Since heart rate and cardiac output are strongly coupled to metabolic rate, Haynes & Inch concluded that the observed falls in heart rate and rectal temperature were fairly well correlated. Although MIS has not been shown markedly to decrease the $O_2$ consumption of V79 and Ehrlich carcinoma cells *in vitro* at concentrations lower than 1 mM, either in the presence or absence of glucose (Biaglow *et al.*, 1978) a 20% reduction of $O_2$ utilization by V79 cells has been reported at a drug concentration of 5 mM (Durand *et al.*, 1978). Our observations on the effect of i.p. administration of MIS on body-core temperature in the absence of anaesthetic indicate that the effect of the drug *in vivo* may be more complex than the data obtained *in vitro* may indicate.

In general, mice have poor temperature regulation, due mainly to their relatively large surface/volume ratio (Bernstein, 1966). No significant changes in body temperature have been reported in cancer patients treated with MIS. However, a clinical incidence of peripheral sensory polyneuropathy has been reported (see introductory paragraph). One possible connection between the lack of evident neurotoxicity in mice after MIS administration and the reported neurotoxicity in human patients may be that, in the mouse, the physiological response of reduction of core temperature may protect the animal against the effect of the toxic chemical species involved in the induction of neurotoxicity.

We are grateful to Professor T. W. Clarkson and Dr R. M. Sutherland for support and many stimulating discussions. We would also like to thank Mrs Laura Nelson for typing this manuscript so competently.

### REFERENCES

BASMAJIAN, J. V. & STECKO, G. (1962) A new bipolar electrode for electrophysiology. *J. Appl. Physiol.*, **17**, 849.

BERNSTEIN, S. E. (1966) Physiological characteristics. In *Biology of the Laboratory Mouse*, 2nd Edition. Ed. E. L. Green. Toronto: McGraw-Hill. p. 337.

BIAGLOW, J. E., NYGAARD, O. F. & GREENSTOCK, C. L. (1974) Redox reactions of anoxic radiosensitizers (Abstr.), *Radiat. Res.*, **59**, 158.

BIAGLOW, J. E., GREENSTOCK, C. L. & DURAND, R. E. (1978) Effects of sensitizers on cell respiration: 1. Factors influencing the effects of hypoxic cells radiosensitizers on oxygen utilization of tumour and cultured mammalian cells. *Br. J. Cancer*, **37** (Suppl. III), 145.

DISCHE, S., SAUNDERS, M. I., LEE, M. E., ADAMS, G. E. & FLOCKHART, I. R. (1977) Clinical testing of the radiosensitizer Ro-07-0582: Experience with multiple doses. *Br. J. Cancer*, **35**, 567.

DURAND, R. E., BIAGLOW, R. E. & GREENSTOCK, C. L. (1978) Effects of sensitizers on cell respiration: III. The effects of hypoxic cell radiosensitizers on oxidative metabolism and the radiation response of an *in vitro* tumour model. *Br. J. Cancer*, **37** (Suppl. III), 150.

DURAND, R. E., BIAGLOW, R. E. & SUTHERLAND, R. M. (1976) Hypoxic radiosensitizers and cellular respiration. *Br. J. Radiol.*, **49**, 567.

FOSTER, J. L., FLOCKHART, I. R., DISCHE, S., GRAY, A. J., LENOX-SMITH, I. & SMITHEN, C. E. (1975) Serum concentration measurements in man of the radiosensitizer Ro-07-0582: Some preliminary results. *Br. J. Cancer*, **31**, 679.

FOWLER, J. R., ADAMS, G. E. & DENEKAMP, J. (1976) Radiosensitizers of hypoxic cells in solid tumours. *Cancer Treatment Rev.*, **3**, 227.

HAYNES, M. J. & INCH, W. R. (1976) Some pharmacological aspects of multiple dose metronidazole in C3H/HeJ mice. *Int. J. Radiat. Oncol. Biol. Phys.*, **1**, 1125.

HIRST, D. E., VOJNOVIC, B., STRATFORD, I. J. & TRAVIS, E. L. (1978) The effect of the radiosensitizer misonidazole on motor nerve conduction velocity in the mouse. *Br. J. Cancer*, **37** (Suppl. III), 237.

KOGELNIK, H. D., MAYER, J. J., JENTZSCH, K. & 6 others (1978) Further clinical experiences of a Phase I study with the hypoxic cell radiosensitizer misonidazole. *Br. J. Cancer*, **37** (Suppl. III), 281.

MCEWAN, L. M. (1956) The effect on the isolated rabbit heart of vagal stimulation and its modification by cocaine, hexamethonium and ouabain. *J. Physiol.* (*Lond.*), **131**, 678.

SAUNDERS, M. I., DISCHE, S., ANDERSON, P. & FLOCKHART, I. R. (1978) The neurotoxicity of misonidazole and its relationship to dose, half-life, and concentration in the serum. *Br. J. Cancer*, **37** (Suppl. III), 268.

SEARLE, A. J. F. & WILLSON, R. L. (1976) Metronidazole (Flagyl): Degradation by the intestinal flora. *Xenobiotica*, **6**, 457.

URTASUN, R. C., BAND, P. R., CHAPMAN, J. D., RABIN, H., WILSON, A. F. & FRYER, C. G. (1977) Clinical Phase I study of the hypoxic cell radiosensitizer Ro-07-0582, a 2-nitromidazole derivative. *Radiology*, **122**, 801.

URTASUN, R. C., CHAPMAN, J. D., FELDSTEIN, M. L. & 6 others (1978) Peripheral neuropathy related to misonidazole: Incidence and pathology. *Br. J. Cancer*, **37** (Suppl. III), 271.